# Tripolar versus bipolar ablation: insights into lesion growth and geometry using a novel ablation approach for therapy-refractory ventricular arrhythmias

**DOI:** 10.1038/s41598-026-48782-y

**Published:** 2026-04-18

**Authors:** Fabian Bahlke, Edison Abdiu, Emily Schultz, Nico Erhard, Florian Englert, Alexander Duda, Hannah Krafft, Miruna Popa, Jan Syväri, Madeleine Tydecks, Alex Tunsch Martinez, Theresa Reiter, Marta Telishevska, Eva Koops, Sarah Lengauer, Gabriele Hessling, Isabel Deisenhofer, Raphael Spittler

**Affiliations:** 1 Department of Electrophysiology, TUM University Hospital German Heart Center, Lazarettstr. 36, 80636 Munich, Germany; 2 Abbott Medical, Minneapolis, MN USA; 3 Department of Cardiology II – Electrophysiology, Center of Cardiology, University Medical Center Mainz, Langenbeckstr. 1, 55131 Mainz, Germany

**Keywords:** Radiofrequency ablation, Bipolar ablation, Lesion formation, Ablation of ventricular tachycardia, Therapy-refractory ventricular tachycardia, Cardiology, Interventional cardiology

## Abstract

Efficacy of bipolar radiofrequency ablation (BA) is often limited by high impedance between the two ablation catheters in cases of therapy-refractory ventricular tachycardias. Tripolar ablation (TA) is a novel approach, adding a dispersive return electrode to BA. The study aimed to investigate differences in lesion growth and geometry in BA and TA using variable power (20–50W) during power-controlled, irrigated radiofrequency ablation on cross-sections of porcine heart preparations in an ex-vivo model. Using high-resolution imaging at one-second intervals, 1694 measurements in 40 lesions were analyzed regarding lesion geometries and ablation parameters. Baseline impedance was lower in TA (213.1 ± 20.6 Ω vs. 242.7 ± 23.6 Ω, *p* < 0.001) and distinct differences in lesion geometry were present. Specifically, TA produced deeper and wider lesions at the active catheter compared to the return catheter, resulting in trapezoidal lesions in contrast to rectangular lesions in BA. All lesions reached transmurality; however, lesion width continued to increase in BA and TA after transmurality had been achieved. The majority of steam pops (16/18) occurred above 40W without differences in BA and TA. In cases of high baseline impedance, TA using an additional dispersive return electrode seems effective. In this experimental setup, TA creates trapezoidal lesions in contrast to rectangular lesions in BA without an associated increase in the risk of steam pops.

**Supplementary Information:**

The online version contains supplementary material available at 10.1038/s41598-026-48782-y.

## Introduction

Ablation of ventricular tachycardia or premature ventricular complexes using radiofrequency (RF) energy remains challenging if the arrhythmogenic substrate is located deep in the myocardium inaccessible by an endocardial approach^1^. Bipolar ablation (BA) is an alternative strategy in these situations and data on its application and safety has been published in recent years^1–4^. In conventional unipolar ablation, the RF catheter applies current that flows toward a dispersive return electrode positioned at the patient’s skin^5,6^. Conversely, in bipolar ablation, the current flows directly to the tip of an opposed return catheter^5,7^. With advantages in technology, bipolar ablation can be set up quickly using dedicated adapters^7^, allowing BA to deliver energy more efficiently within a localized area. However, a significant risk of steam pops has been reported^8,9^.

The primary advantage of BA is its ability to achieve transmural lesions improving outcome even in difficult cases, such as hypertrophic cardiomyopathy or arrhythmias from the left ventricular summit, interventricular septum, crux cordis or papillary muscle^1,2,5,10,11^. Notably, BA is often considered after optimization strategies for unipolar ablation have been exhausted, including modulation of dispersive return electrode positioning and using half-saline irrigation, as these approaches aim to enhance lesion formation by modifying current distribution^6,12,13^. This highlights the use of BA in treating arrhythmias that unipolar ablation failed to achieve success^1,14^. In this context, previous work by Kany et al. also suggested that sequential unipolar before BA can improve lesion formation in challenging ablation procedures^15^.

Despite these advantages, BA is also challenging in clinical practice^15^. High impedance values can significantly impede its efficacy^4,16^. In some cases, this issue can be solved by increasing the surface of the return catheter, as described by Younis et al.^17^ Nevertheless, even ideal positioning of the return catheter does not overcome the problem of an impedance of 250 Ω and more, particularly in BA targeting the left ventricular summit from the left ventricular outflow tract and the coronary sinus which may limit ablation efficacy. In this setting, an impedance mismatch, defined as an imbalance in the electrical load between the active and return catheter due to heterogeneous electrode-tissue-coupling, can restrict effective current delivery and result in insufficient lesion creation^18^.

This study evaluates lesion growth and geometry with BA in comparison to a novel approach incorporating an additional dispersive return electrode, which results in a tripolar ablation (TA) configuration. In contrast to recent trials using multipolar mapping catheters as return catheter^19,20^, this study focusses on real-time changes in lesion geometry and ablation parameters.

## Methods

### Ex-vivo-model

**Fig. 1:**
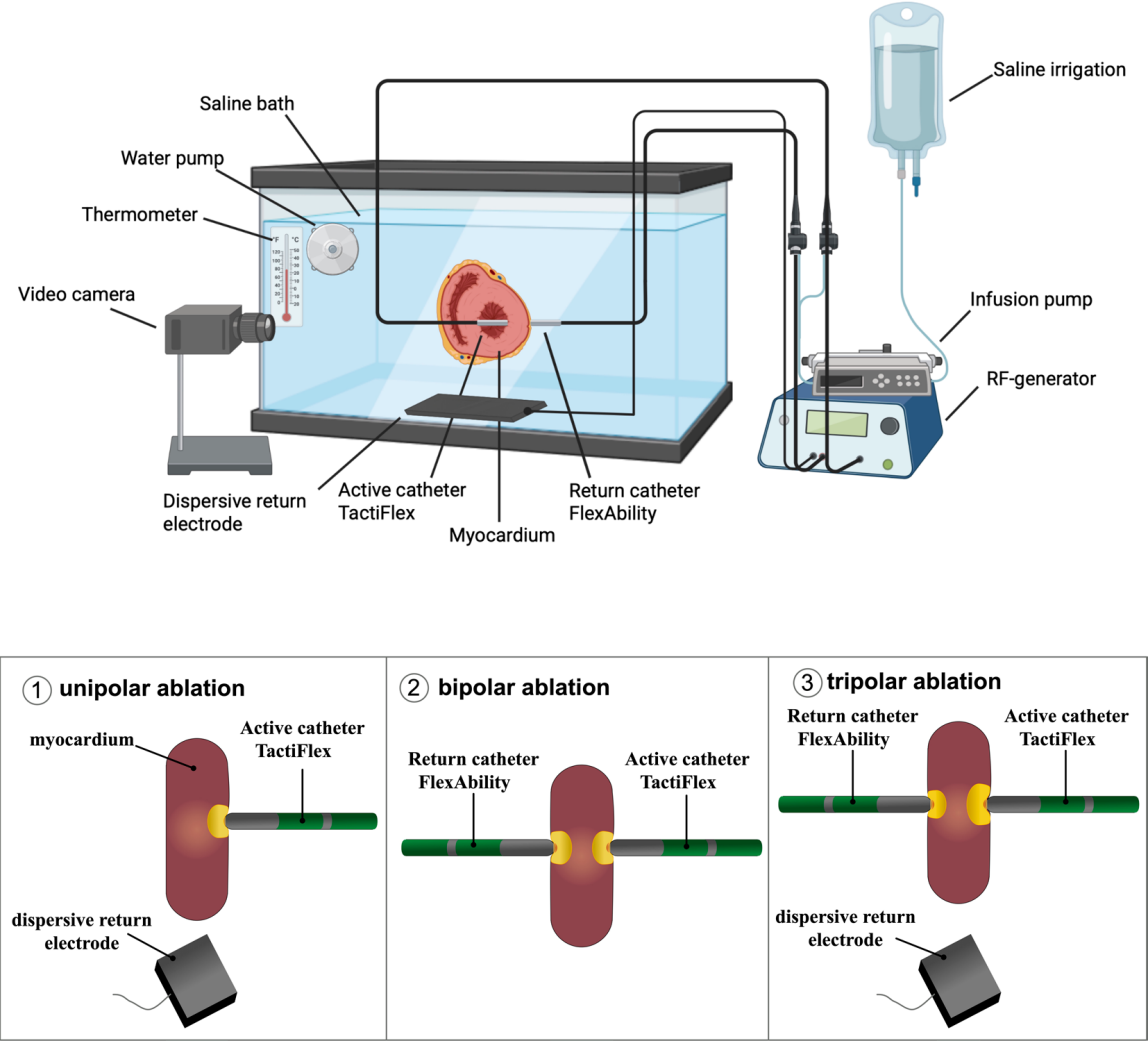
Upper panel: Schematic illustration of the experimental setup containing a saline-filled container, cross-sectioned porcine myocardium, two ablation catheters, a radiofrequency (RF)-generator, a thermometer, a dispersive return electrode for tripolar ablation, an infusion pump, saline infusion and a video camera. Bipolar and tripolar ablation setups were used. Lower panel: Experimental setups for unipolar, bipolar and tripolar ablation. For bipolar ablation, two ablation catheters were placed on opposing sites of the tissue cross-sections. Tripolar ablation was performed by adding a dispersive return electrode to the bipolar setup. Parts of the figure were created with biorender.com.

**Fig. 2:**
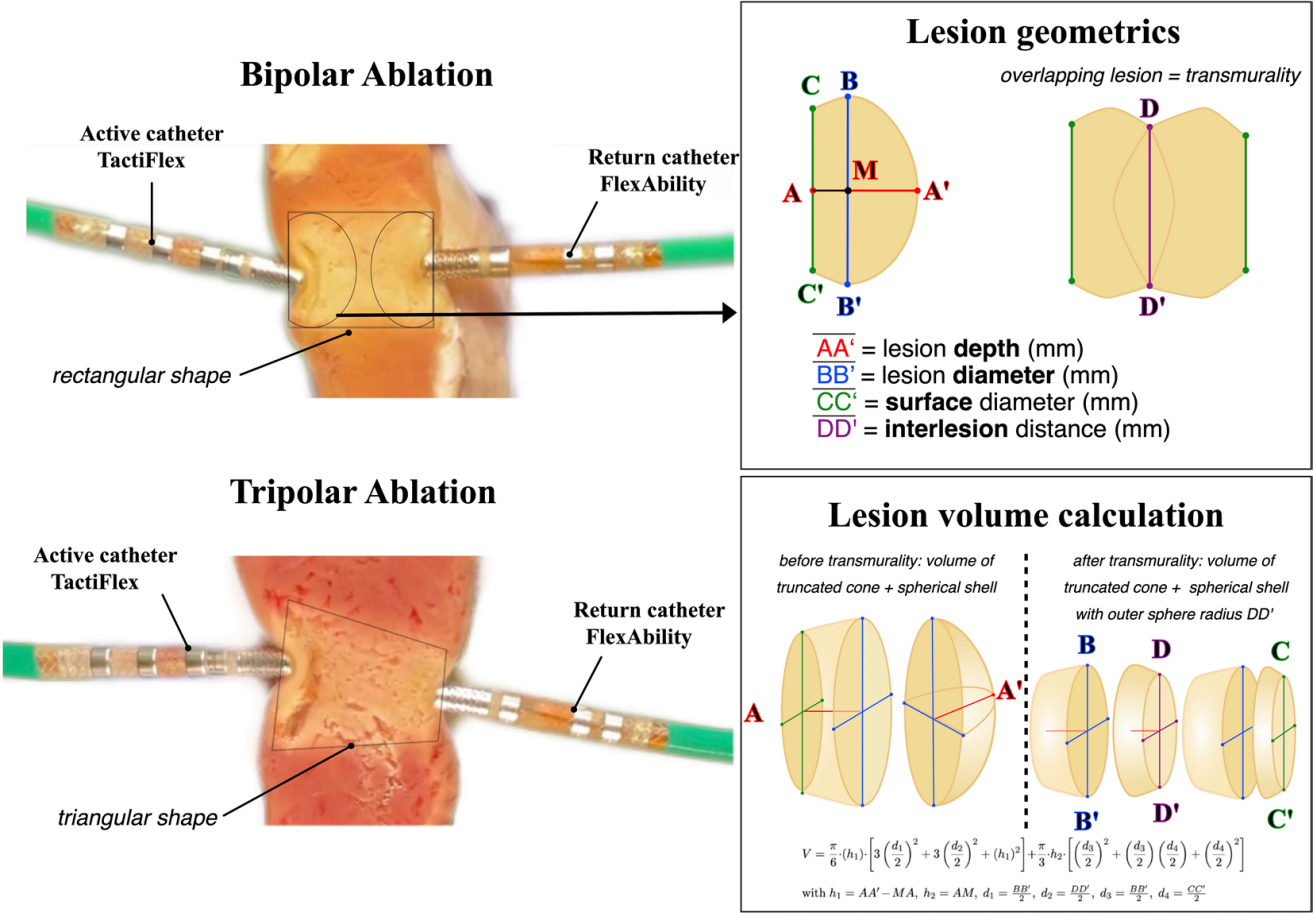
Left panel: Comparison of lesion geometry created by bipolar and tripolar ablation in myocardial cross-section. Right panel: Schematic illustration of lesion parameters measurements and lesion volume calculation. The following parameters were measured for each lesion at both the active and the passive catheter sites: lesion depth (distance from point (A) to (A’)), lesion diameter (distance from point (B) to (B’)), depth from the surface to the diameter (distance from point (A) to (M)) and diameter at myocardial surface (distance from point (C) to (C’)) were measured. The overlapping diameter of the opposing lesion is illustrated as distance from point (D) to (D’) (‘kissing lesion’). Lesion volume was calculated using these geometric parameters by modeling the lesion as the sum of a truncated cone and a spherical shell, or a spherical segment when lesion overlap was present.

BA and TA were performed in a previously validated ex-vivo-model imitating clinical conditions^21^. A total of five porcine hearts were used and processed within a maximum of 48 h after slaughter. Lesions were preferentially created in the intraventricular septum or the left ventricular walls. However, the right ventricle was used when the left ventricle was excessively hypertrophied and did not allow adequate imitation of clinically relevant conditions. The swine myocardium was fixed in a permanently circulated bath. Using a heated thermostat, the conditions were temperature controlled (37 °C) and the baseline system impedance was kept continuously at 120Ω in the saline bath alone with a saline concentration of 1.72 g/l. To maintain this impedance target in the saline bath, saline or distilled water was added if necessary. In contrast to the model by Lacko et al.^21^, our model used porcine cross-sections to establish a real-time visualization of lesion growth by recording the whole ablation process in high-definition quality, as described before by our^10,22^ group (Fig. 1). For BA setup, two irrigated ablation catheters were placed perpendicularly to the myocardium with a contact force (CF) range of 10–15 g (Fig. 2). The BA setup was previously described by Futyma et al.^7^ The active catheter (TactiFlex, Abbott, Minneapolis, Minnesota, USA) was positioned at one side of the cross-section and the return catheter (Flexability, Abbott, Minneapolis, Minnesota, USA) at the opposite site of the myocardial surface. Swine myocardium and both catheters were fixed by clamping and manual stabilization. Irrigation was set to 18 ml/min for both ablation catheters. For TA, an additional dispersive return electrode (Fiab, Italy; Model F7905W/V, surface area 136 cm^2^) was added to the BA setup in the second dispersive electrode port (Fig. 1), enabling comparative evaluation of lesion characteristics of both approaches. The dispersive return electrode was placed below the porcine preparations and its position was constant during all applications.

### Ablation protocol

Overall, five power settings (20W, 30W, 40W and 50W) and two ablation configurations (BA and TA) were tested in this study, aiming to create 5 lesions in each group. Ablation was stopped in case of a steam pop or after 20 s after achieving transmurality. Lesions from different power settings and ablation configurations were distributed across all five hearts to minimize confounding by heart-specific factors. As described above, all lesions were recorded with a high-resolution camera to collect information on lesion growth at each second of RF delivery. Impedance measurements were obtained using the standard impedance monitoring algorithms implemented in the EnSite X system (Abbott, Minneapolis, Minnesota, USA) and no local impedance measurements were performed. The system applies a low-amplitude alternating current at a fixed frequency to determine tissue impedance, independent of RF energy delivery. Impedance, current, CF and catheter position acquisition was performed continuously with system-defined sampling intervals of 20 ms. The same measurement configuration was used for both bipolar and tripolar ablation setups.

### Lesion measurements

Lesion measurements were assessed by visual change in myocardial tissue color, calibrated against the 4 mm ablation catheter tip (IC Measure Program, Version 3.0.0.521). For each lesion, the following parameters were measured (see Fig. 2, including Figure legend): lesion depth (distance from the midpoint of the surface (A) to the deepest point of the lesion (A’) in mm), lesion diameter (distance from the upper vertex (B) to the lower vertex (B’) in mm), depth from the surface to the diameter (distance from the midpoint of the surface (A) to the middle of the diameter (M) in mm) and the lesion diameter at the myocardium surface (distance from the surface upper point (C) to the surface lower point (C’) in mm). Subsequently, the overlapping diameter (interface) of the two opposite lesions was also quantified (distance from the upper intersection point (D) to the lower intersection point (D’) in mm).

Lesion metrics at the active catheter were defined as depth (active catheter), diameter (active catheter), surface-depth (active catheter), surface-diameter (active catheter) and at the return catheter as depth (return catheter), diameter (return catheter), surface-depth (return catheter) and surface-diameter (return catheter). Using these metrics, several ratios were defined for analysis of lesion geometrics:

- Ratio of lesion depth at the active catheter and return catheter:
  $$\text{Rdepth }=\frac{depth \text{(active catheter)}}{depth \text{(return catheter)}}$$
- Ratio of maximum diameter at the active and return catheter:
  $$\text{Rdiameter }=\frac{diameter \text{(active catheter)}}{diameter \text{(return catheter)}}$$
- Ratio of the surface diameter at the active and return catheter:
  $$\text{Rsurface }=\frac{\text{surface diameter (active catheter)}}{\text{surface diameter (return catheter)}}$$

Transmurality was defined as the point at which lesions at the active and return catheters made contact. The occurrence of steam pops was observed through both auditory and visually means, including the occurrence of sudden tissue distortion. Continuous recording ensured that all data were available even in lesions with steam pops at the end.

Lesion volume was calculated using the model of a truncated cone combined with a spherical segment, which best matched the observed lesion geometry:

$$V=\frac{\uppi }{6}\cdot \left({h}_{1}\right)\cdot \left[3{\left(\frac{{d}_{1}}{2}\right)}^{2}+3{\left(\frac{{d}_{2}}{2}\right)}^{2}+{\left({h}_{1}\right)}^{2}\right]+\frac{\uppi }{3}\cdot {h}_{2}\cdot \left[{\left(\frac{{d}_{3}}{2}\right)}^{2}+\left(\frac{{d}_{3}}{2}\right)\left(\frac{{d}_{1}}{2}\right)+{\left(\frac{{d}_{1}}{2}\right)}^{2}\right]$$

$${h}_{1}=A{A}{\prime}-MA, {h}_{2}=AM, {d}_{1}=\frac{B{B}{\prime}}{2}, {d}_{2}=\frac{D{D}{\prime}}{2}, {d}_{3}=\frac{{CC}{\prime}}{2}$$

V denotes the lesion volume; h_1_ is defined as the distance from the midpoint of the lesion diameter to the deepest extent of the lesion; h_2_ represents the distance from the midpoint of the lesion diameter at the myocardial surface to the midpoint of the lesion diameter; d_1_ denotes radius of the lesion; d_2_ denotes the radius of the inner interlesion surface; d_3_ denotes the radius of the outer lesion at the myocardial surface.

In order to make the time for transmurality comparable, we normalized the increase of the lesion depth to the distance of the two electrodes as follows:

$$\left[\frac{\text{distance between active and return catheter} - \text{remaining lesion depth}}{\text{distance between active and return catheter}}\right]$$

All experiments were conducted in accordance with institutional guidelines, the German Animal Welfare Act and the European Directive. No in vivo experiments were performed, and all tissue samples were obtained post-mortem.

### Statistical analysis

Continuous variables are presented as mean with standard deviation. Between-group differences in continuous variables were tested using the Student’s t-test or Mann–Whitney U test, as appropriate. A test for normal distribution was performed using the Shapiro–Wilk test. Between-group differences in categorical variables were analyzed with the Fisher’s exact test. Categorical variables were compared using odds ratios with 95% confidence intervals. Variance was tested using an F-test. Correlations were calculated using the Spearman’s rank correlation coefficient. Values are expressed as mean ± standard deviation. All significance tests were two-tailed, with the null hypothesis rejected at *p* < 0.05. Receiver operator characteristic (ROC) analysis with area under the curve (AUC) was performed to determine the impedance cut-off for steam pops. Data points in graphs represent mean value at discrete time points with 95% confidence interval error bars. To visualize temporal propagation, a non-parametric regression curve was fitted using a generalized additive model (GAM) with limited spline flexibility.

The ratio of lesion volumes, depth and diameter between the active and passive catheter were calculated on a per-time-point basis, and the mean and standard deviation of this ratio were determined across all shared time points.

The statistical analysis was conducted using the R programming language (R Foundation for Statistical Computing, R Development Core Team, Vienna, Austria).

## Results

### Baseline characteristics of the experimental setup

In total, 40 lesions were created, comprising 5 lesions at each power setting (20–50W) and ablation configuration (BA and TA). On average, the thickness of the myocardial tissue was 19.3 ± 4.2 mm in the BA group and 21.2 ± 3.5 mm in the TA group (*p* = 0.14). After catheter placement and applying CF of 10–15 g, the minimal distance of both catheters was lower in the BA group compared to the TA group (17.8 ± 3.9 vs. 19.8 ± 3.5 mm, *p* = 0.09). Mean CF in all lesions was 13.0 ± 6.2 g with no difference between BA and TA (12.8 ± 5.9 g vs. 13.1 ± 6.5 g; *p* = 0.61). Baseline impedance was significantly lower in the TA group (242.7 ± 23.6Ω vs. 213.1 ± 20.6Ω; *p* < 0.001). In TA, the reduction of the baseline impedance by adding the dispersive return electrode was 59.3 ± 14.4Ω. Transmurality was achieved in all lesions after a mean RF duration of 22.0 ± 10.7 s in BA and 29.3 ± 16.4 s in TA (*p* = 0.18). Results of lesion geometry and impedance values during BA and TA are presented in Table 1.

**Table 1:** Comparison of myocardial, electrical and lesion parameters in bipolar and tripolar ablation at baseline and at transmurality.

	Bipolar ablation (n = 20)	Tripolar ablation (n = 20)	*p*-value
Minimal myocardial thickness [mm]	17.8 ± 3.9	19.8 ± 3.5	0.09
Time to transmurality [s]	22.0 ± 10.7	29.3 ± 16.4	0.18
Baseline impedance [Ω]	242.7 ± 23.6	213.1 ± 20.6	< 0.001
Impedance at transmurality [Ω]	175.4 ± 12.8	160.9 ± 17.8	0.004
Impedance drop [Ω]	67.4 ± 22.1	52.2 ± 15.4	0.016
Relative impedance drop [%]	27.3 ± 7.0	24.4 ± 6.3	0.168
Current [mA]	442.5 ± 83.3	461.7 ± 97.3	0.51
Maximum depth (active catheter) [mm]	9.0 ± 2.0	10.8 ± 2.5	0.008
Maximum depth (return catheter) [mm]	8.7 ± 2.1	8.9 ± 2.0	0.78
Maximum diameter (active catheter) [mm]	10.9 ± 3.1	10.9 ± 2.5	0.99
Maximum diameter (return catheter) [mm]	10.3 ± 2.9	9.0 ± 2.0	0.06

### Temporal changes in lesion formation and impedance

**Fig. 3:**
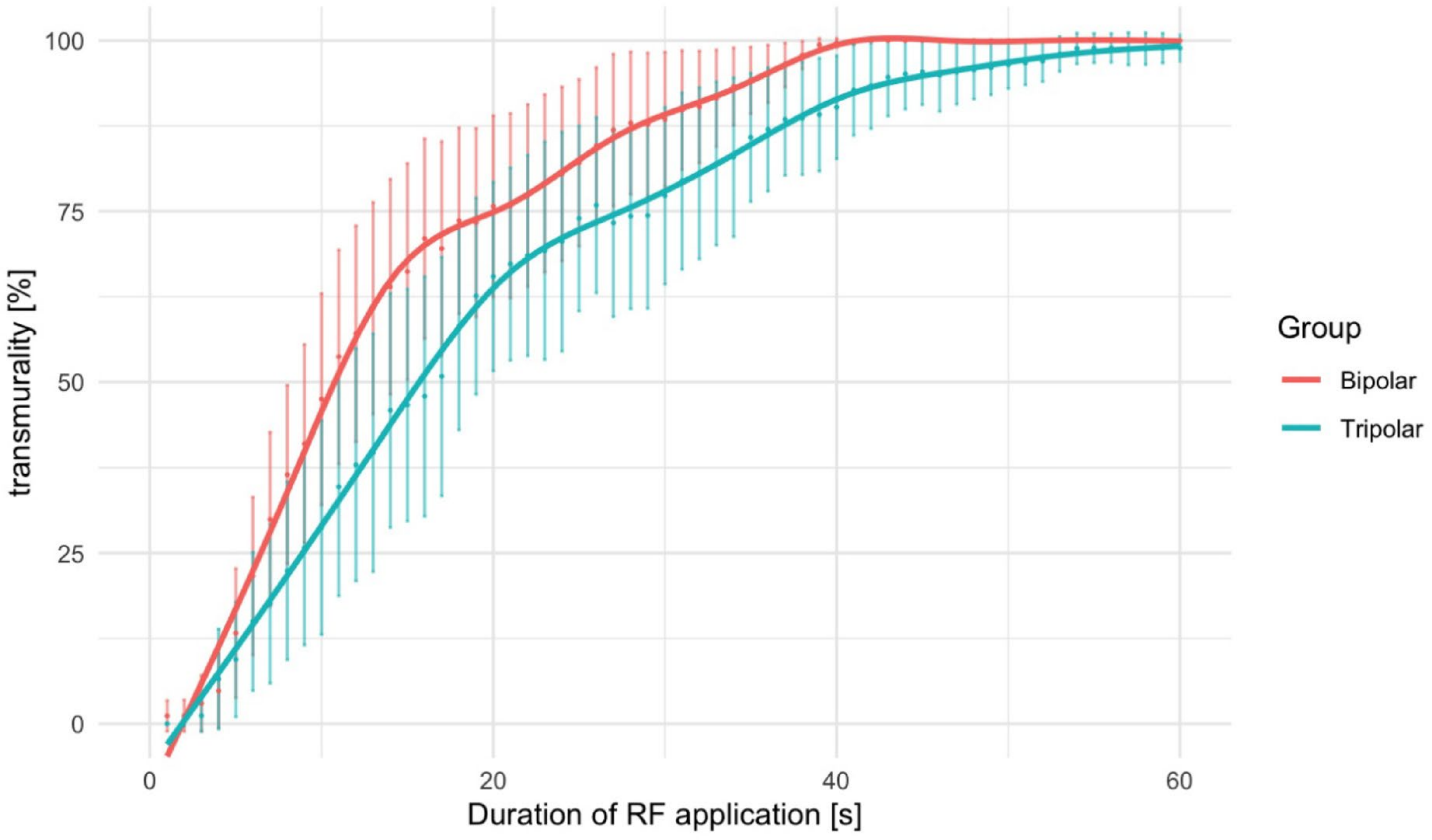
The mean relative transmurality (transmurality in relation to the initial myocardial thickness at the onset of radiofrequency ablation, measured as the distance between the active and return catheters) of bipolar ablation compared to tripolar ablation. Bipolar ablation demonstrates a shorter time to transmurality and tripolar ablation approach requires more time to reach transmurality. Overall, the thermal propagation is not linear and slower in TA than in BA. Every data point represents mean value (including all power settings) with error bars indicating 95% confidence intervals. The solid lines represent GAM-based regression curves (non-parametric) to illustrate temporal progression. RF = radiofrequency.

**Fig. 4:**
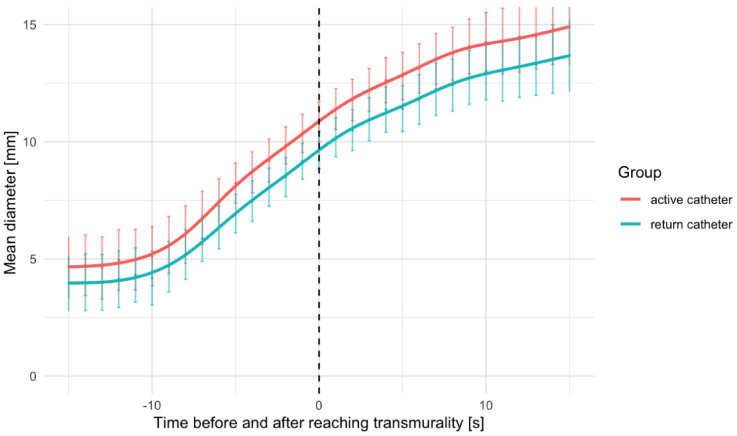
Mean maximal diameter at the active (blue) and return (blue) catheter before and after reaching transmurality. Every data point represents mean value with error bars indicating 95% confidence intervals. The solid lines represent GAM-based regression curves (non-parametric) to illustrate temporal progression.

Figure 3 illustrates the remaining lesion depth until transmurality during BA and TA across all lesions. BA demonstrates a shorter time to 50% and complete (100%) transmurality compared to TA. At the individual power levels, transmurality occurred after 28.7 ± 12.4 s at 20W, after 36.4 ± 17.5 s at 30W, after 19.4 ± 4.2 s at 40W and after 18.1 ± 11.8 s at 50W (Supplementary Fig. S1). Interestingly, lesion diameter at both the active catheter and the return catheter continues to expand even after transmurality has been achieved (Fig. 4).

**Fig. 5:**
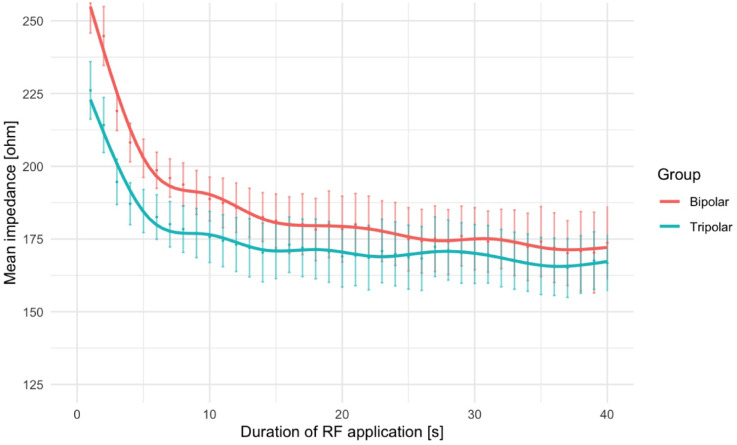
Mean impedance during RF application in bipolar (red) and tripolar ablation (blue). The use of an additional dispersive return electrode in tripolar ablation results in an initially lower mean impedance compared to bipolar ablation. Notably, the impedance level in bipolar ablation does not reach the same low levels observed in tripolar ablation during the RF application Every data point represents mean value with error bars indicating 95% confidence intervals. The solid lines represent GAM-based regression curves (non-parametric) to illustrate temporal progression. RF = radiofrequency.

Impedance dropped rapidly during the initial seconds of ablation under both conditions and stabilized after 15 s (Fig. 5). Thereafter, mean impedance values converged and from 15 s onward, no significant difference was observed between both groups (180.35 ± 21.61Ω in BA vs. 170.37 ± 22.58Ω in TA; *p* = 0.16). The average impedance drop after RF onset was lower in TA (67.4 ± 22.1Ω vs. 52.2 ± 15.4Ω; *p* = 0.016; Table 1).

### Differences in lesion geometry and volume

#### Lesion shape and active-return asymmetry in tripolar ablation

BA leads to more symmetrical, rectangular-shaped lesions, whereas TA results in lesions with a trapezoidal-like shape in cross section.

Lesions at the active and return catheter during BA exhibited comparable width and depth (Rdepth and Rdiameter of 1.0 ± 0.2). In contrast, TA showed pronounced lesion asymmetry, with a 30% greater depth and 20% larger diameter at the active catheter (Rdepth 1.3 ± 0.4 and Rdiameter 1.2 ± 0.2; both *p* < 0.01).

#### Lesion geometry at the time of transmurality

**Fig. 6:**
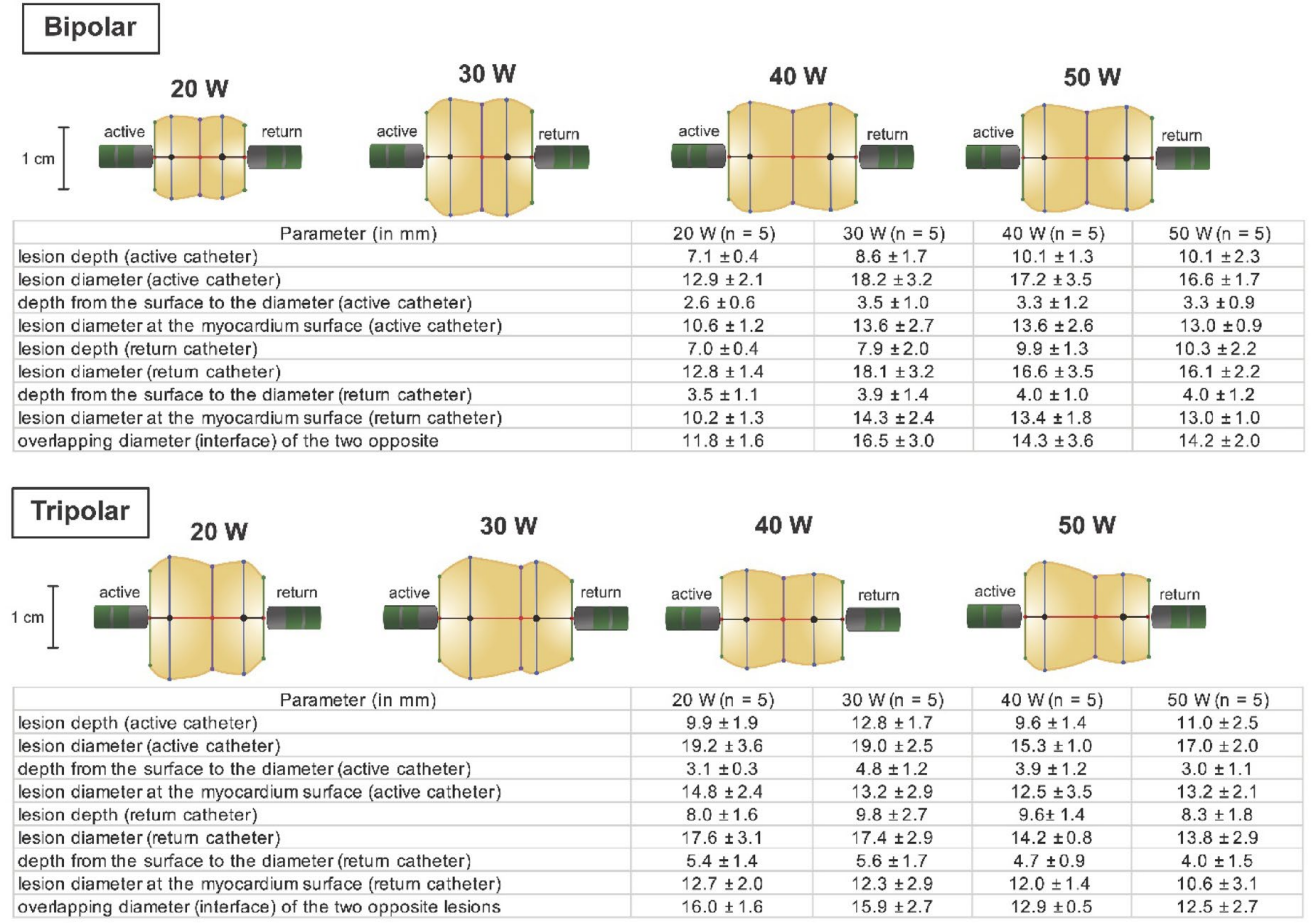
Scaled representation of lesions generated by bipolar and tripolar ablation at different power settings at the time transmurality was achieved. Overall lesion size varies due to differences in myocardial thickness and ablation duration until transmurality was achieved. Bipolar ablation produced more evenly distributed lesion depths, whereas tripolar ablation resulted in a deeper and wider lesion at the active catheter. W: watt.

Upon achievement of transmurality, lesion depth at the active catheter was significantly greater in TA than in BA (10.79 ± 2.17 mm vs. 8.95 ± 1.96 mm; *p* = 0.008). Additionally, the point of lesion fusion (“kissing lesion”) was closer to the return catheter in TA, reflected by a higher Rdepth (1.25 ± 0.30 vs. 1.03 ± 0.12, *p* = 0.006) and a shorter kissing lesion diameter in TA (5.01 ± 3.50 mm vs. 8.59 ± 3.21 mm; *p* = 0.002). Lesion metrics for the different power settings are summarized in Fig. 6. Differences in lesion characteristics were observed between BA and TA. All power settings in the TA setting show the shift from the interlesion line towards the return electrode.

#### Lesion volume distribution

**Fig. 7:**
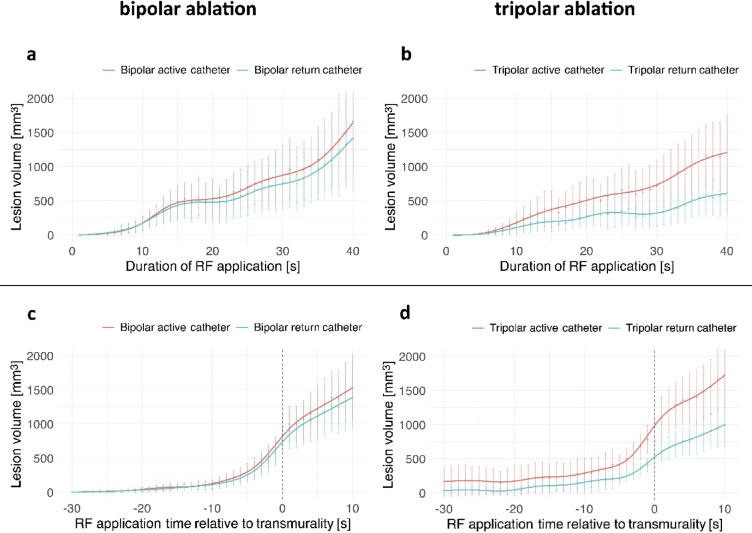
Mean lesion volume at the active catheter (red) and return catheter (blue) during bipolar ablation (left, **a** and **c**) and tripolar ablation (right, **b** and **d**). In the upper panels (**a**, **b**), the x-axis shows time to radiofrequency (RF) onset (absolute RF application duration). In the lower panels (**c**, **d**), time is aligned to transmurality (transmural lesion at timepoint 0, black dotted line, negative values indicate time before transmurality). Every data point represents mean value with error bars indicating 95% confidence intervals. The solid lines represent GAM-based regression curves (non-parametric) to illustrate temporal progression. RF = radiofrequency.

In BA, lesion volume was comparable between the active and return catheter (ratio: 1.17 ± 0.44). In contrast, TA resulted in larger lesion volumes at the active catheter (ratio: 2.19 ± 1.46, *p* = 0.007). Once transmurality was achieved, 52.9 ± 5.9% of the total lesion volume was located at the active catheter in BA compared with 63.9 ± 11.7% in TA (*p* = 0.007; Fig. 7). In more detail, the ratio between lesion volumes at the active and passive catheter sites in tripolar ablation was 2.4 ± 0.7 at 20W, 3.1 ± 1.4 at 30W, 1.2 ± 0.2 at 40W and 2.0 ± 0.9 at 50W. In contrast, BA resulted in a nearly symmetric lesion distribution, with a volume ratio close to 1 (Supplementary Fig. S2).

Total lesion volumes did not differ between BA and TA when achieving transmurality (BA: 1760 ± 1088mm^3^ vs. TA: 1576 ± 943mm^3^; *p* = 0.57).

### Safety and occurrence of steam pops

During creation of the 40 lesions, 18 steam pops were observed. Of those, 16 steam pops occurred during ablation with 40W and 50W, two steam pops during application of 30W. Mean time to steam pop occurrence was 28.9 ± 14.1 s. All steam pops were noticed after reaching transmurality (mean 9.7 s ± 4.8 s after transmurality). No significant changes were observed in frequency and timing of steam pops between BA and TA (nine steam pops in each setting; time to steam pop 28.3 ± 12.1 s vs. 29.4 ± 16.7 s, *p* = 0.87). Additionally, there were no differences in steam pop frequency at the active and passive catheter in BA and TA. 6 out of 9 steam pops occurred at the active catheter in BA and 7 out of 9 steam pops at the active catheter in TA. The ROC analysis identified an impedance cut-off of 139.5Ω for TA, with a sensitivity of 75% and a specificity of 93.3%. For BA, the cut-off was 156.5Ω, yielding a sensitivity of 88.9% and a specificity of 89.4% (Supplementary Fig. S3).

## Discussion

To the best of our knowledge, this is the first study that systematically evaluates real-time lesion growth in bipolar ablation compared to a novel tripolar ablation approach that incorporates an additional dispersive return electrode. Whereas BA results in lesions with a rectangular-like shape, TA lesions show a trapezoidal-like shape, suggesting an altered current distribution. TA lesion shape might result mainly from the reduced baseline impedance leading to increased current density at the active catheter. Concerning safety, both approaches were comparable regarding steam pops that predominantly occurred using 40W or more.

### Impact of the dispersive return electrode on baseline impedance

Beyond the challenge of ablating deep intramural lesions, high impedance may constitute a limiting factor in distinct clinical situations, particularly during epicardial ablation or ablation within the coronary venous system. Strategies such as using half saline or an 8 mm tip have been proposed to mitigate this issue^14,17^.

However, by adding a dispersive return electrode in the TA setup, baseline impedance was reduced significantly by 59.3 ± 14.4Ω in this experimental setup. This approach appears to be effective and may serve as a bail-out strategy. Since impedance is crucial for current delivery into the tissue^23^, lowering impedance might influence current flow and subsequently lesion growth and geometry. Importantly, dispersive return electrode positioning can also modulate current pathways, thereby potentially affecting lesion formation and geometry in TA^6,24,25^. In clinical scenarios involving high impedance due to impedance mismatch, TA may be considered as a potential option in situation where other ablation strategies have failed. Nonetheless, further trials are needed to directly compare tripolar ablation with the use of half saline or an 8 mm tip to better define their respective roles in clinical practice^13,17^.

### Lesion formation and geometry in bipolar ablation

In BA, lesions at the active and return catheter grow symmetrically with comparable lesions volumes lesion depth and lesion width. Our study contributes three novel insights relevant for practical application:

First, lesion growth is symmetrical in BA throughout the entire RF application under the experimental conditions of the present study. Previous studies, such as Nguyen et al. showed symmetrical lesion formation in bipolar ablation at the end of ablation^11^. Our study observed this symmetry continuously during RF application. This is relevant for clinical use, as lesions can be assumed symmetrical even if the application has to be stopped for safety reasons and transmurality is not achieved, although this assumption may not hold in the presence of epicardial fat or scarred tissue, which can alter the current distribution^15,26^.

Second, after transmurality is achieved, lesion width at the active and return catheter and the diameter of the kissing lesion increase even further in both, BA and TA. This emphasizes the importance of balancing RF duration in clinical use to achieve required lesion diameter while avoiding damage to adjacent structures and occurrence of steam pops.

Impedance during RF ablation declines primarily due to tissue heating and is influenced by several parameters in clinical practice, as blood flow and electrode-tissue-coverage^22,27^. Given the progressive increase in lesion diameter after reaching transmurality, defining a definitive impedance drop threshold to predict transmurality in BA and TA remains challenging, representing the third novel insight in BA. Based on the results, the majority of lesion depth is achieved when impedance decreases at a rate of approximately 0.5–1.0Ω/s. Given the limitations of impedance-based thresholds, incorporating composite ablation indices together with interelectrode distance may provide a more robust approach for detecting transmurality, as Inaba et al. described in a recent publication^28^.

### Changes in lesion geometry in tripolar ablation

In TA, current density is altered by adding a dispersive return electrode. In relation to the lesion at the return catheter, the lesion at the active catheter is deeper and wider, resulting in a higher lesion volume. Additionally, the ‘kissing lesion’ is closer to the return catheter in TA and the diameter of the ‘kissing lesion’ is smaller in TA when transmurality is achieved. This effect was observed in all different power setting. All findings support the observation of trapezoidal-like shaped lesions in TA.

Therefore, adding the dispersive return electrode seems to lower current density at the return catheter. The altered lesion geometry might be useful in clinical practice. In cases of intramural substrate, which might be asymmetrically distributed and closer to the endo- or epicardium^29^, choosing the active catheter at the corresponding site closer to the intramural substrate can optimize TA for a more precise and efficient approach. In addition to its potential for efficacy, TA might enhance safety by minimizing the risk of damage to adjacent structures close to the return catheter. In this context, multipolar setups might also be able to create asymmetrical lesions as described by Fernandes et al., who used an octapolar catheter as return catheter to reach previously inaccessible intramural substrates^20^. Based on the selection of return electrodes, current flow can be modulated. Similarly, Menkovic et al. demonstrated the feasibility of multipolar ablation using a high-density mapping catheter epicardially as return catheter^19^. In both studies, baseline impedance did not differ from that observed in bipolar ablation. Both multipolar and tripolar ablation may be considered in scenarios where asymmetrical lesion formation is desired, although their clinical roles require further translational studies. Future work could also explore the use of tripolar ablation in cases with normal baseline impedance.

### Safety of bipolar and tripolar ablation

A higher incidence of steam pops during ablation was observed in high power settings using 40W and 50W while only two steam pops occurred with 30W. No significant differences in steam pop frequency and timing were observed between BA and TA, as all steam pops occurred after transmurality had been achieved. Consistent with this safety concern, Igarashi et al. also reported an elevated risk for steam pops with more than 45W and John et al. described more steam pops in 40W compared to 30W^8,30^. However, we observed different impedance thresholds for steam pop occurrence between TA and BA, with lower thresholds in TA than in BA.

Transmurality was achieved with all settings, including using 20 and 30W. Recent clinical trials reported a low incidence of steam pops at an average^1,14^ power of 30–36W. Kany et al. also reported low rates of steam pops in an ex vivo model using bipolar ablation at 50W and also combining unipolar ablation (30W) with sequential bipolar ablation (50W)^15^.

In consideration of recent clinical evidence and our results, the use of power levels exceeding 30W in BA and TA might not improve efficacy but could raise relevant safety concerns. Continuous impedance monitoring is crucial to guide safe RF application.

## Limitations

Extrapolation of the ex-vivo model to clinical practice is not entirely possible. Imitation of clinical conditions in the experimental setup cannot completely overcome this issue. Lacko et al.^21^ compared a similar ex-vivo setup with in-vivo conditions and did not observe significant differences. In contrast to our experimental model, in-vivo models are inherently limited to single-point measurements of lesion size at the end of RF application. To the best of our knowledge, solely ex-vivo models can provide real-time visualization of lesion formation and ensure a high degree of catheter stability. Although the experimental setup applied a strictly standardized positioning of the dispersive return electrode, translation to clinical practice is limited as the position of the dispersive return electrode alone can influence lesion growth and geometry^6,24,25^. As continuous CF monitoring was not available with the return catheter, variations in CF are possible. The number of lesions delivered is lower compared to recently published trials^8,22^. By implementing continuous measurement of lesions size and ablation parameters, the number of lesions was reduced, as earlier trials had to create lesions with different RF durations. Multiple lesions were created across all five hearts, with power settings and ablation configurations distributed to reduce within-heart bias; however, analysis was performed at the lesion level without explicit modeling of heart-level clustering, and residual intra-heart correlation cannot be excluded. All lesions were created using two catheters with equal tip size to exclude possible influence of tip size on lesion growth^17^. Frequency of steam pops might be overestimated in ex vivo experiments as perfusion affects impedance and heat dissipation. Despite these limitations, our experiments provide proof of concept and warrant further investigation.

## Conclusions

In cases of high baseline impedance, TA incorporating a dispersive return electrode appears effective in lesion creation despite a modestly longer time required to achieve transmurality compared to BA. TA reduces baseline impedance and modifies lesion geometry to create wider and deeper lesions at the active catheter. Visualization of lesion formation revealed symmetrical and rectangular lesions in BA and asymmetrical and trapezoidal lesions in TA. Safety of TA seems comparable to BA in our experimental setup but the altered lesion geometry needs to be considered in a clinical setting to avoid collateral damage. Using power settings < 40 W for both BA and TA might significantly decrease the occurrence of steam pops. Overall, TA may have potential as an adjunctive tool in selected cases of therapy-refractory ventricular arrhythmias. However, further translational work is required before its integration into clinical practice.

## Supplementary Information

Below is the link to the electronic supplementary material.

Supplementary Material 1

## Data Availability

The data set used and/or analyzed during the current study are available from the corresponding author on reasonable request.

## Abbreviations

BA: Bipolar ablation
CF: Contact force
RF: Radiofrequency
TA: Tripolar ablation
