# Supplementary material for: Tripolar versus bipolar ablation: insights into lesion growth and geometry using a novel ablation approach for therapy-refractory ventricular arrhythmias
Source: Sci Rep. 2026 Apr 18;16:12739. doi: 10.1038/s41598-026-48782-y (PMC13091789; doi:10.1038/s41598-026-48782-y)
Supplement: Supplementary file 1 — Supplementary Material 1 [file 41598_2026_48782_MOESM1_ESM.docx]

**Supplementary Material**

**
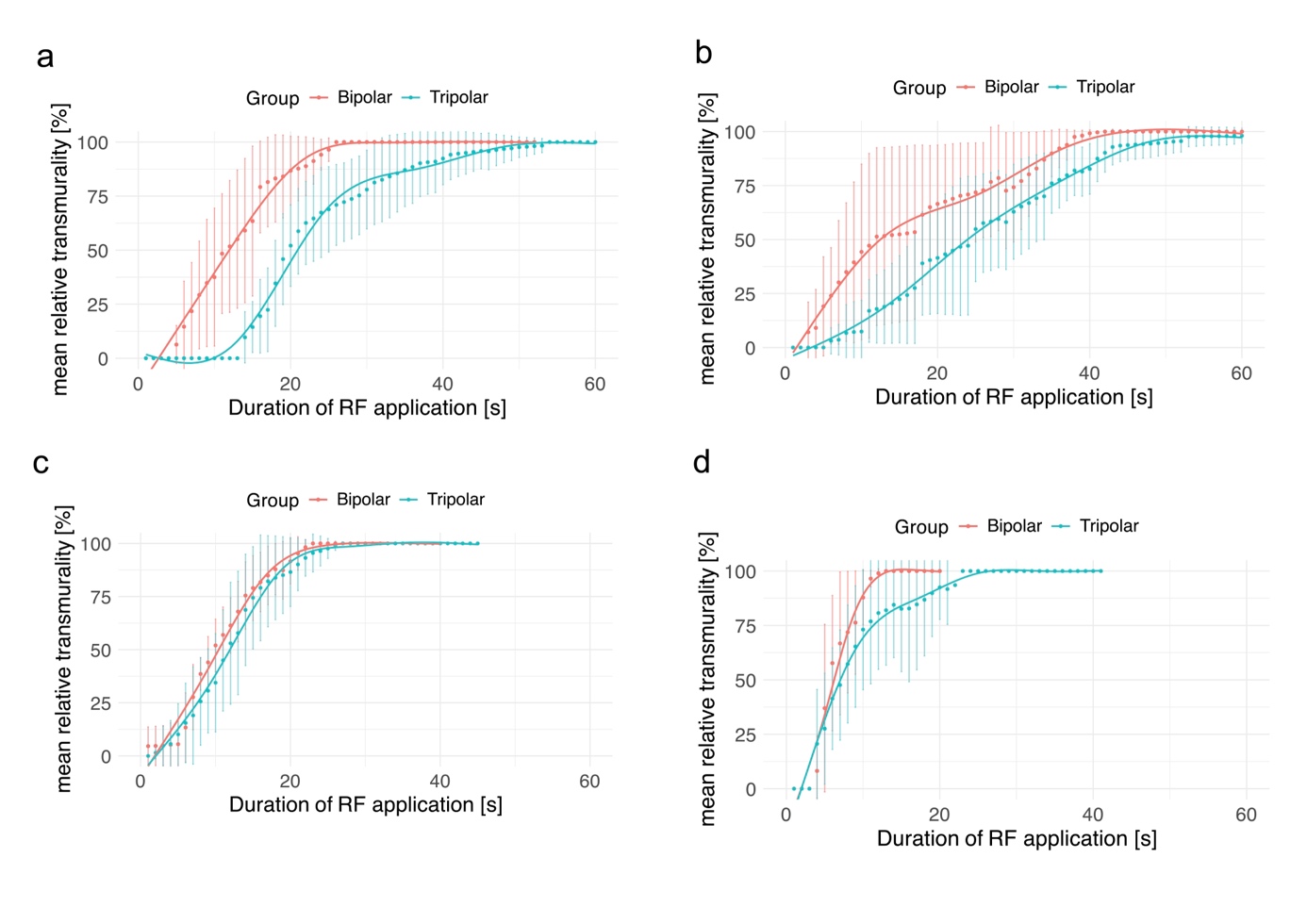
**

Supplementary Figure S1. Relative transmurality for different power settings: Panel a: 20W; Panel b: 30W; Panel c: 40W; Panel d: 50W. Data points represent mean values with error bars indicating 95% confidence interval and regression curve for illustration of progression. RF = radiofrequency.

**
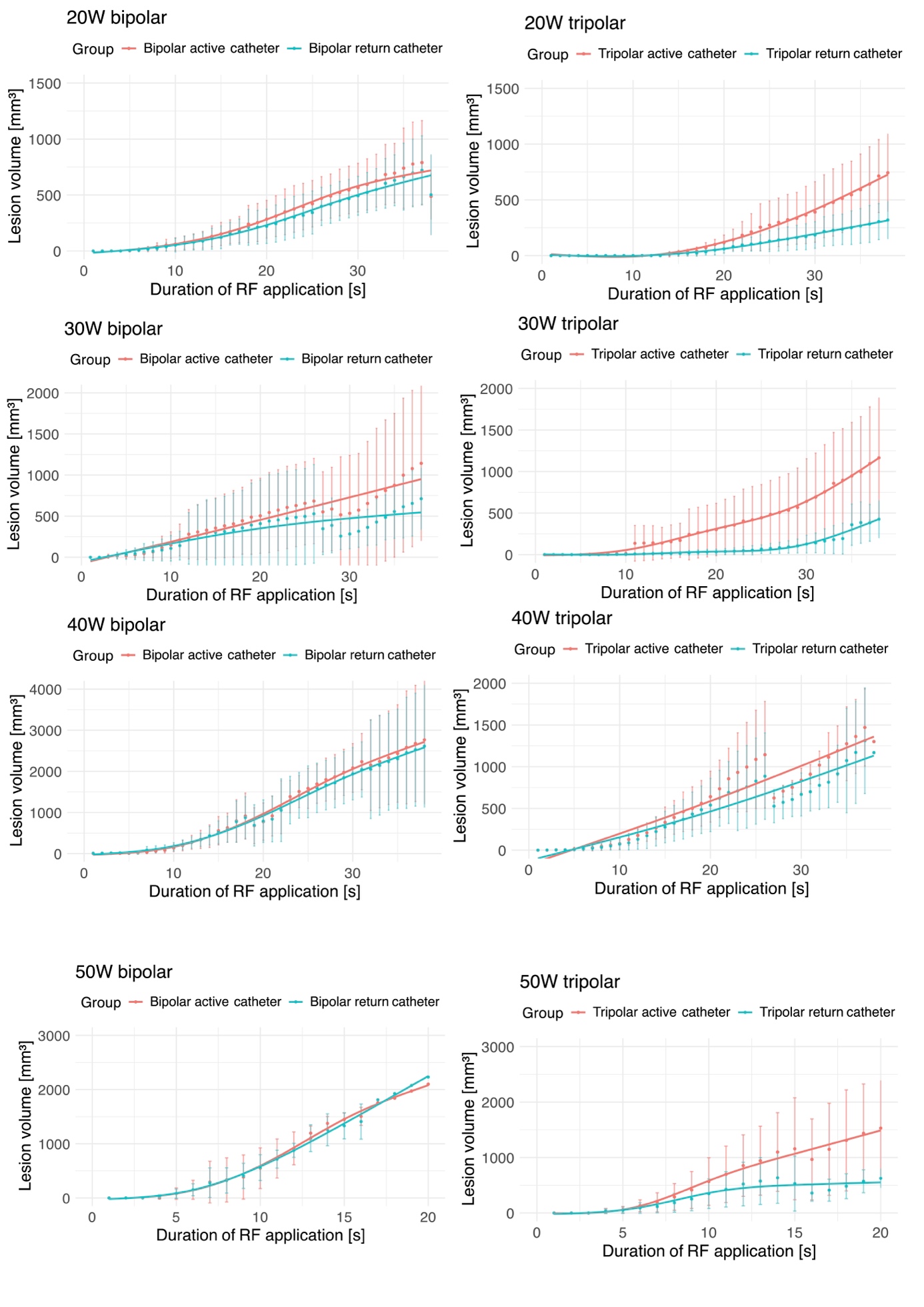
**

Supplementary Figure S2. Lesion volume in each power setting. Lesion volume was calculated using the formula described in the Methods section. The figure illustrates lesion volume over time at active catheter site (red) and the return catheter site (blue). Bipolar ablation is shown on the left, and tripolar ablation on the right. From top to bottom, panels compare lesion volumes for 20W, 30W, 40W, 50W. Every data point represents a mean value with error bars indicating 95% confidence interval and regression curve for illustration of progression. Volume ratios were calculated for every power setting separately; The volume ratio for tripolar ablation was 2.4 ± 0.7 at 20W, 3.1 ± 1.4 at 30W, 1.2 ± 0.2 at 40W and 2.0 ± 0.9 at 50W and for bipolar ablation was 1.13 ± 0.1 at 20W, 1.4 ± 0.8 at 30W, 1.0 ± 0.1 at 40W and 1.0 ± 0.1 at 50W. RF = radiofrequency.


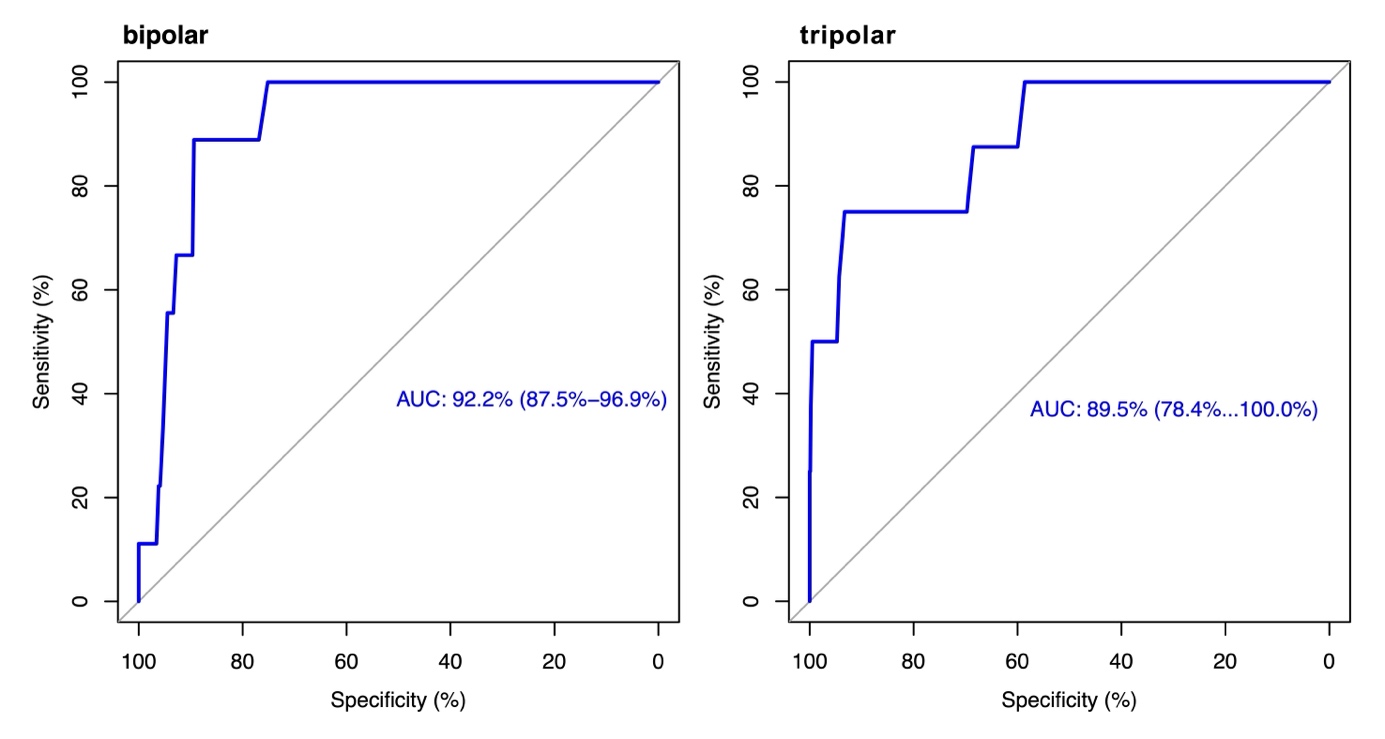


Supplementary Figure S3. Receiver operating characteristics (ROC) of steam pop and impedance for tripolar (right) and bipolar (left) ablation setting. The cut-off for impedance was 139.5Ω with a sensitivity of 75% and a specificity of 93.3% for the tripolar ablation and for the bipolar ablation with a cut-off 156.5Ω with a specificity of 89.4% and a sensitivity of 88.9%. AUC: area under the curve.
